# Care managers’ confidence in managing home-based end-of-life care: a cross-sectional study

**DOI:** 10.1186/1471-2318-13-67

**Published:** 2013-07-01

**Authors:** Maiko Watanabe, Noriko Yamamoto-Mitani, Masakazu Nishigaki, Yuko Okamoto, Ayumi Igarashi, Miho Suzuki

**Affiliations:** 1 Department of Adult Nursing/Palliative Care Nursing, Division of Health Sciences & Nursing, Graduate School of Medicine, The University of Tokyo, 7-3-1 Hongo, Bunkyo-ku, Tokyo 113-0033, Japan; 2 Department of Gerontological Nursing, Graduate School of Health Care Sciences, The Tokyo Medical & Dental University, 1-5-45 Yushima, Bunkyo-ku, Tokyo 113-0034, Japan

**Keywords:** End-of-life, Home Care, Confidence, Older People, Care Managers, Professional Background

## Abstract

**Background:**

There are increasing occasions for care managers (CMs) to manage end-of-life (EOL) situations for older persons at home, in Japan. However, many CMs report anxiety, difficulties and low confidence in managing such care, although confidence is considered a significant determinant of professional performance. This study examined the confidence of CMs at managing home-based EOL situations and its factors.

**Methods:**

Participants of this cross-sectional study were CMs from 1,200 homecare agencies in Japan, which were systematically sampled from a national database. Participants were asked about their overall confidence in managing home-based EOL situations, as well as their demographic, professional and agency characteristics. Multiple logistic regression analysis was conducted to examine the factors associated with CM confidence levels.

**Results:**

Valid responses were obtained from 458 participants (response rate, 39.4%). Among the respondents, 81.0% (n = 371) were female; mean age 49.2 years old (standard deviation = 8.8). Their professional backgrounds included nurses (28.2%), care workers (49.8%), social workers (10.9%), and home attendants (6.1%). Approximately 70% of CMs expressed some level of confidence in managing home-based EOL situations. Multiple logistic regression analysis showed that being confident was significantly associated with having a nursing license (OR: 2.71, 95% CI: 1.26–6.19) and having an additional work responsibility other than being a CM, such as working as a homecare nurse or a home attendant (OR: 2.78, 95% CI: 1.06–4.74). Higher confidence levels were more frequently reported among those who had multiple experiences with EOL situations, compared with those who had none, or only one experience: OR=2.60 (95% CI 1.26–5.50) for those with 2-3 cases; OR=7.12 (3.21–16.56) for those with 4-10 cases; OR = 33.67 (8.14–235.19) for those with 11 cases and over.

**Conclusions:**

These results suggest that CMs with direct, hands-on experience with EOL care, or who have managed multiple EOL cases, tended to be confident at managing home-based EOL situations. Given that the number of nurses working as CMs is decreasing, further research is needed to explore what support CMs need to increase their confidence, especially when the CMs do not have nursing licenses and/or experience with EOL situations.

## Background

Home-based end-of-life (EOL) care means providing personal and palliative care using healthcare and social services at a person’s home, during the last days of his/her life [1]. It is considered a desirable form of care for persons nearing the end of their lives from both a quality-of-life and economic point of view [2]. People who received home-based EOL care tend to die with a better functional status, psychological well-being and cognitive status than those who have not had this type of care. Bereaved families who receive home-based EOL care also tend to report more favorable experiences than families without such care [3]. Further, the cost of home-based EOL care tends to be lower than that of hospital and/or hospice-based care [4,5].

In reality, however, only 13% of all deaths recorded in Japan actually occurred at home in 2011 [6], even though over half of the general public, and patients with a terminal illness, prefer to stay home until the end of their lives [7]. One major reason for this discrepancy is the lack of effective management of healthcare professionals and social services, and overall care management involved with home EOL care. Care management in EOL situations is generally more complicated than that of other situations because of numerous EOL-related conditions, which are often difficult to control, such as delirium [8].

In Japan, there is a new professional called a “care manager” (CM) who is responsible for service organization and management of each case. This role has been introduced under the Long Term Care Insurance (LTCI) System, which covers long-term nursing care and social services for persons aged 65 years and over, as well as those between 40 and 64 years with specific age-related diseases [9]. Under the LTCI, elderly persons are assisted by a CM with their service use and care management. CMs are responsible for developing and managing an individual care service plan, which should be adjusted on a regular basis based on changes to the client’s situation [9].

There is wide variability among CMs with regards to their background education and training. There are more than 20 professional groups such as “care workers”, social workers, and nurses, who are allowed to apply for a CM national license if they have 5 years’ worth of clinical experience. A 2012 survey reported that as many as 66% of CMs possessed a background license for “care worker” [10]. “Care worker” is a professional role similar to “home health aides (HHAs)” in the United States, who provide personal care to those with care needs. Unlike HHAs, care workers are considered independent professional care providers with an independent national licensure, although currently the pathway to achieve licensing is variable; ranging from only personal care clinical experience to having a bachelor degree [11,12]. The proportion of CMs with a nursing license in 2012 was just 8.9%; this figure is decreasing under a climate of a general shortage of nurses in long-term care settings.

One difficulty that CMs often experience relates to managing EOL situations [13]. The 44-hour-long training program undertaken to become a CM, which involves lectures and care planning practices, does not support them to acquire the necessary abilities and skills for managing EOL scenarios. For example, the programs do not cover how to monitor deteriorating physical conditions over time, or how to change care plans according to the patient’s physical state. As a consequence, many CMs are left feeling unsure about their EOL care competence; their confidence levels often have room for improvement [13,14].

Past research suggests that confidence is essential for any healthcare professional to function optimally [15-18]. Confidence is “a belief in one’s own abilities, self-assurance” [19]. Exploring the factors of CMs’ confidence to manage home-based EOL situations is important so that they can meet the growing expectations around effective home-based EOL care.

There are a limited number of studies about the confidence of CMs in managing home-based EOL situations. A qualitative study that explored perceptions around the facilitators and barriers to advance care planning processes in the United States, found that previous experience with care planning had a positive impact on the CM’s confidence [20]. Another study found that having a professional nursing background was significantly associated with having confidence in case management of older persons in need of medical care, compared with having a professional care worker background [14]. In each of these studies, the experience and/or situations were not specified. Thus, the purpose of this study was to examine the confidence of CMs in managing home-based EOL situations, and its factors, with a focus on the CM’s past experience and professional characteristics.

## Methods

### Study design and participants

A cross-sectional survey was conducted. In October 2011, a self-administered questionnaire was mailed to 1,200 agencies. The agencies were systematically sampled from 30,548 agencies held in a public national database of residential services and in-home service agencies under the LTCI system. Any one CM at each care manager agency could participate in the study. Agencies were excluded in areas under the Disaster Relief Act that were affected by the Tohoku Region Pacific Coast Earthquake and Northern Nagano Prefecture Earthquake in March 2011. A postcard was mailed to each participant after the nominated deadline to remind them to return the questionnaire.

The research protocol was examined and approved by the ethics committee of the Japan Visiting Nursing Foundation. Returning a completed questionnaire was deemed to represent the participant’s consent to be part of the study.

### Measures

The questionnaire consisted of items relating to confidence in managing home-based EOL situations, and the participant’s agency, demographic and professional characteristics.

#### Confidence in managing home-based EOL situations

Overall confidence in managing home-based EOL situations was assessed using a single item with a four-point Likert scale, which asked: “Suppose your client requests home EOL care, would you be confident in managing home-based EOL situation for him/her until s/he dies?” The responses to the item were dichotomized into two categories: confident (“confident” and “somewhat confident”) and not confident (“not very confident” and “not confident”).

#### Agency characteristics

Agency characteristics were collated from five aspects: 1) agency ownership; 2) presence of other healthcare facility/facilities in the same organization; 3) the number of full time-equivalent CMs; 4) the number of clients per month; and 5) the number of clients who had died at home within the last 6 months.

#### Care manager characteristics; demographic and professional characteristics

The age and gender were recorded for the participants’ demographic characteristics. Their professional characteristics were assessed using six aspects: 1) professional background (previous role); 2) years of experience in their previous profession before becoming a CM; 3) additional responsibilities other than care management in the same agency (e.g. working also as a homecare nurse); 4) years since gaining a CM license; 5) the number of EOL cases experienced as a CM; and 6) previous experience of working in medical facility.

### Statistical analysis

Univariate analyses of all items were undertaken, followed by bivariate analyses using simple logistic regression to examine the relationship between confidence and all other variables. Descriptive analyses revealed that the distribution of two continuous variables were highly skewed: the number of clients who had died at home within the last 6 months in an agency, and the number of EOL cases experienced by the CM; as a result these were categorized into four group. All continuous variables were examined for their association with confidence as a dichotomous variable using bivariate analyses. A linear relationship was not observed, either on the scatter plots or by correlational analysis for the following three variables, which were used as categorical variables: the number of clients in the agency per month, years since gaining their CM license, and the number of years they worked in their previous profession. Variables related to CM confidence at *p* <0.05 from the simple logistic regression analyses were forced entered into a multiple logistic regression analysis, with confidence being the dependent variable. Multi-collinearity was examined by variance inflation factor. All statistical analyses were run with R version 2.15.1.

## Results

Of the 1,200 distributed questionnaires, 21 were returned because of an unknown address. Overall, 476 questionnaires were returned (40.4% response rate), but 18 were excluded because they had no data about CM confidence levels. The final number of participants was 458 (39.4% valid response rate).

### Participant characteristics and their confidence in managing home-based EOL situations

Table 1 outlines the participant characteristics and their overall confidence in managing home-based EOL situations. The mean age of the participants was 49.2 ± 8.8 years; about 80% were female. Nearly half of the participants (49.8%) had a care worker license, 10.9% had a social worker license, and 28.2% had a nursing license (registered nurse, licensed practical nurse, or public health nurse). A quarter of the participants had an additional responsibility other than care management. Approximately 23% had managed none, or only one EOL situation. Over 70% of the participants reported some level of confidence in managing home-based EOL situations: confident (40.6%) and somewhat confident (33.8%).

**Table 1:** Participant characteristics (N= 458)

		**n (%) or Mean ± SD**
**Agency characteristics**		
Agency ownership	Profit corporation	168 (36.7)
	Social welfare corporation	141(30.8)
	Healthcare corporation	87 (19.0)
	Others	65 (14.2)
Other health-care facility(ies)	Yes	125 (27.3)
in the same organization	Hospitals	91 (19.9)
	Home nursing	74 (16.2)
Number of FTE care managers	0–1.4	116 (25.3)
	1.5–2.0	48 (10.5)
	2.1–3.5	76 (16.6)
	3.6 and over	68 (14.8)
Number of clients / 1 month	0–32	104 (22.7)
	33–59	105 (22.9)
	60–97	107 (23.4)
	98 and over	104 (22.7)
Number of clients who had died at home / 6 months	0–1	293 (64.0)
	2 and over	152 (33.2)
**Care manager characteristics**		
Age (years)		49.2 ± 8.78
Gender	Female	371(81.0)
Professional background	RN, LPN, or PHN	129 (28.2)
	Care worker	228 (49.8)
	Social worker	50 (10.9)
	Home attendant	28 (6.1)
	Others	72 (15.7)
Years of experience in background profession	0–7	180 (39.3)
	8–12	126 (27.5)
	13 and over	148 (32.3)
Additional responsibility other than care management	Yes	116 (25.3)
Years since gaining a care manager license	0–3	62 (13.5)
	4–5	96 (21.0)
	6–8	138 (30.1)
	9 and over	140 (30.6)
Number of end-of-life cases experienced	0–1	106 (23.1)
	2–3	106 (23.1)
	4–10	143 (31.2)
	11 and over	68 (14.8)
Previous experience of working in medical facility	Yes	286 (62.4)
Confidence in managing home-based end-of-life care	Not confident	25 (5.5)
	Not very confident	92 (20.1)
	Somewhat confident	155 (33.8)
	Confident	186 (40.6)

Missing values were removed.

*FTE* Fulltime Equivalent.

*RN* Registered Nurse, *LPN* Licensed Practical Nurse, *PHN* Public Health Nurse.

### Simple logistic regression analyses for their confidence in managing home-based EOL situations

Simple logistic regression analyses revealed that having a nursing license (Odds Ratio (OR) 3.54, 95% Confidence Interval (CI) 1.99–6.72) and having an additional responsibility other than care management (OR 1.88, 95% CI 1.10–3.22) were significantly associated with being confident in managing home-based EOL situations. Participants who had multiple experiences of managing home-based EOL situations were more likely to be confident (2–3 cases: OR 2.22, 95% CI 1.26–3.97; 4–10 cases, OR 5.43, 95% CI 3.00–10.12; 11 cases and over: OR 30.63, 95% CI 8.91–192.83), compared with participants who had experienced one case, or none (Table 2).

**Table 2:** Simple logistic regression analysis predicting the likelihood of participants with confidence (N=436)

		**Odds ratio**	**95% Confidence interval**
**Agency characteristics**			
Other health-care facility(ies) owned by the same organization [yes = 1, no = 0]		1.22	(0.88–1.75)
Number of FTE care managers	0–1.4	1.00	[reference]
	1.5–2.0	1.01	(0.59–1.75)
	2.1–3.5	1.67	(0.94–2.99)
	3.6 and over	3.79***	(1.90–8.03)
Number of clients / 1 month	0–32	1.00	[reference]
	33–59	0.96	(0.54–1.70)
	60–97	2.16*	(1.15–4.12)
	98 and over	2.54**	(1.33–5.00)
Number of clients who had died at home / 6 months		2.65***	(1.62–4.48)
[2 and over = 1, 0–1 = 0]			
**Care manager characteristics**			
Age (years)		1.04*	(1.01–1.07)
Gender [female = 1, male = 0]		1.71*	(1.03–2.80)
Professional background [yes =1, no = 0]	RN, LPN, or PHN	3.54***	(1.99–6.72)
	Care worker	0.77	(0.50–1.17)
	Social worker	1.09	(0.56–2.24)
	Home attendant	0.75	(0.33–1.88)
	Others	0.60	(0.35–1.04)
Years of experience in background profession	0–7	1.00	[ reference ]
	8–12	2.04*	(1.19–3.57)
	13 and over	2.38**	(1.42–4.09)
Additional responsibility other than care management [no=1, yes=0]	1.88 *	(1.10–3.22)	
Years since gaining a care manager license	0–3	1.00	[reference]
	4–5	2.11*	(1.07–4.17)
	6–8	3.03***	(1.59–5.84)
	9 and over	3.82***	(2.01–7.32)
Number of end-of-life cases experienced			
	0–1	1.00	[ reference ]
	2–3	2.22*	(1.26–3.97)
	4–10	5.43***	(3.00–10.12)
	11 and over	30.63***	(8.91–192.83)
Previous experience of working in medical facility [yes = 1, no = 0]		1.79***	(1.15–2.77)

* p < 0.05. ** p < 0.01. *** p < 0.001.

*FTE* Fulltime Equivalent.

*RN* Registered Nurse, *LPN* Licensed Practical Nurse, *PHN* Public Health Nurse.

### Multiple logistic regression analysis for their confidence in managing home-based EOL situations

When multi-collinearity was examined among all the independent variables, multi-collinearity existed between the number of clients in the agency per month and the number of FTE care managers, and also between professional background and previous experience of working in a medical facility. Therefore, the number of FTE care managers and previous working experience were omitted from the final model. Table 3 presents the results of a multiple logistic regression analysis. The significant predictors for the CM’s confidence in managing home-based EOL situations were: older age (OR 1.04, CI 1.01–1.08), having a nursing license (OR 2.71, CI 1.26–6.19 [reference: not having nursing license]), having 8–12 years of experience in their previous/background profession (OR 2.18, CI 1.07–4.57), and having an additional work responsibility other than care management (OR 2.78, CI 1.06–4.74). Confidence was more frequently reported among those who had multiple experiences of managing EOL situations, than among those who had none, or only one experience (2–3 cases: OR 2.60, CI 1.26–5.50; 4–10 cases: OR 7.12, CI 3.21–16.56), 11 cases and over: OR 33.67, CI 8.14–235.19) [reference: not having multiple EOL experiences]).

**Table 3:** Multiple logistic regression analysis predicting the likelihood of participants with confidence (N = 436)

		**Odds ratio**	**95% Confidence interval**
**Agency characteristics**			
Number of clients / 1 month	0–32	1.00	[ reference ]
	33–59	0.90	(0.42–1.90)
	60–97	2.09	(0.91–4.94)
	98 and over	2.30	(0.95–5.79)
Number of clients who had died at home / 6 months [ 2 and over = 1, 0-1 = 0]		1.08	(0.52–2.29)
**Care manager characteristics**			
Age (years)		1.04*	(1.01–1.08)
Gender [ female = 1, male = 0]		1.05	(0.50–2.24)
Professional background [ nurse=1, non-nurse=0]		2.71*	(1.26–6.19)
Years of experience in background profession	0–7	1.00	[ reference ]
	8–12	2.18*	(1.07–4.57)
	13 and over	1.72	(0.83–3.63)
Additional responsibility other than care management [ no=1, yes=0 ]		2.78**	(1.06–4.74)
Years since gaining care manager license	0–3	1.00	[ reference ]
	4–5	0.80	(0.31–2.00)
	6–8	0.95	(0.38–2.36)
	9 and over	0.94	(0.34–2.55)
Number of end-of-life cases experienced	0–1	1.00	[ reference ]
	2–3	2.60*	(1.26–5.50)
	4–10	7.12***	(3.21–16.56)
	11 and over	33.67***	(8.14–235.19)
Goodness-of-fit statistics^1^ (*χ*^2^, p)		4.73	0.79

1 The Hosmer and Lemeshow Goodness-of-Fit Test.

Nurse includes registered nurse, licensed practical nurse, or public health nurse.

^*^p < 0.05. ^**^p < 0.01. ^***^p < 0.001.

## Discussion

This study investigated the confidence of CMs in managing home-based EOL situations and its factors. Our participants’ agency and demographic characteristics were similar to that of a national survey from Japan [21]. The observed response rate in this study was not high, although it was higher than expected when compared with a previous study [22].

In this study, approximately 70% of CMs were confident in managing EOL situations. This rate is relatively high when compared to a previous study that explored the confidence of general practitioners (GPs) in delivering home-based EOL care, where the proportion of GPs that felt confident was 56.6% [23]. This difference may be due to the wording of the questions; in our study CMs were asked about their confidence in general, while the GPs were asked about their confidence about taking responsibility. It may also be due to differences in the extent of responsibility for care provision; CMs do not usually engage in direct care of the client, while GPs are often responsible for direct symptom management.

Consideration is needed about what can be done to help CMs with low confidence. Participants who had nursing backgrounds were more likely to be confident than those who did not; this result is consistent with previous studies [14,24]. Knowledge about the physiological aspects of EOL situations and their previous hospital experience with EOL care may explain why more CMs with nursing licenses had confidence, than those without such a license. This suggests that EOL cases may be best managed by nurse CMs. However, CMs with a nursing license are already scarce [25] and their numbers are decreasing. In the general climate of nursing shortages within long-term care settings, it is very plausible that CMs without a nursing license will continue to take responsible for the majority of EOL cases. Specific assistance is needed that are tailored for CMs without nursing licenses.

Having an additional work responsibility, other than care management, was also associated with having greater confidence. Although the details of their additional positions were not collected, they were most likely to be nurse or care worker roles [25]. The CMs who had additional positions may have had more opportunities to directly observe and provide care to patients and their families. This may mean that having the confidence to be a CM requires hands-on experience in delivering home-based EOL care. Further studies to examine the impact of having an additional work responsibility among CMs are needed. Educational programs that enable CMs to experience direct contact with clients/families may have a positive impact on CM confidence; this also deserves further examination and study.

The CMs who had more experience with home-based EOL case management were more likely to be confident. A study of Danish GPs also showed a significant positive relationship between the number of home-based EOL experiences and their confidence in having principal responsibility for the client’s care [23]. Another study reported that previous experience with care planning facilitated improved confidence in case managers [20]. Gaining greater hands-on experience with managing EOL situations may improve skill levels for engaging with clients about advanced planning and their future care needs. In summary, training should include relevant and practical examples of managing home-based EOL circumstances.

The relationship between the confidence of CMs and the number of years worked in their background profession since gaining their CM license was not linear in this study. [25] Yoshie et al. concluded that CMs who were more experienced, often reported greater difficulties at managing cases, and were often assigned more difficult cases [14]. The study of GPs also showed that the number of years in general practice was not associated with their level of confidence when dealing with home-based EOL care. CMs with extensive experience in their background profession are often considered to be highly skilled. However, they may actually practice as CMs without enough official training, specifically with regard to the management of cases with medical problems. As a result, educational opportunities should be provided to all CMs, regardless of the length of time they have been engaged in care management.

The overall results of this study suggest there is a need for educational and practical training for those who lack the confidence for managing home-based EOL cases. In particular, those without nursing as their background profession, additional work responsibilities, or multiple experiences of managing EOL situations. Opportunities are needed for these CMs to learn about EOL management, especially about managing the physiological/biological changes at the EOL.

There are four major limitations to this study. First, the study’s generalizability is limited because the participants were a single CM, from each care manager agency, and the response rate was under 50%. Second, the confidence in managing home-based EOL situations was evaluated using an overall, single item. Confidence is likely to be composed of multiple domains, such as physiological/biological knowledge, case management skill, and/or EOL communication. The areas of knowledge and skills that cause low confidence levels should be identified. Third, because of the cross-sectional design of this study, no inference about causal relationships can be made. A longitudinal study is needed to examine whether the confidence levels in CMs is a result of, or results in, for example, more experience with EOL care. Fourth, the CMs’ performance was not measured in this study; we cannot determine that CMs’ higher confidence levels translated into improved performance [26]. An evaluation method for CM performance has not been established; further studies are needed to better understand the relationship between CM confidence and CM performance regarding their management of home-based EOL care.

## Conclusions

In this cross sectional survey about the confidence of CMs in managing home-based EOL situations, we found that having a nursing background, an additional work-responsibility other than care management, and broad experience with home-based EOL management, were all positively associated with confidence in this group. This suggests that physiological/biological knowledge of the dying process, having direct, hands-on experience with EOL care, and accumulating a range of experiences, may be important for enabling CMs to be confident at managing home-based EOL situations. In future studies researchers should explore what CMs need to improve their confidence, especially those without nursing license and/or experience with EOL care. In particular, the educational needs of CMs should be explored, and the effectiveness of educational interventions on home-based EOL management should be examined.

## Abbreviations

CM: Care manager; EOL: End of life; FTE: Full-time equivalent; GP: General practitioner; LTCI: Long-term care insurance.

## Competing interests

The authors declare that they have no competing interests.

## Authors’ contributions

YMN took the primary role in conceiving and conducting the survey as well as supervising the data analysis and manuscript development. WM conceived and performed data analysis, interpreted the data, and drafted the manuscript. SM assisted data interpretation, jointly developed and edited the manuscript. NM helped manage the data. OY and IA jointly conceived and conducted the survey and primary data analyses. All authors read and approved the final manuscript.

## Pre-publication history

The pre-publication history for this paper can be accessed here:

http://www.biomedcentral.com/1471-2318/13/67/prepub
